# Dynamic Maritime Traffic Pattern Recognition with Online Cleaning, Compression, Partition, and Clustering of AIS Data

**DOI:** 10.3390/s22166307

**Published:** 2022-08-22

**Authors:** Yuanqiang Zhang, Weifeng Li

**Affiliations:** 1 Faculty of Maritime and Transportation, Ningbo University, Ningbo 315211, China; 2 Navigation College, Dalian Maritime University, Dalian 116026, China

**Keywords:** intelligent transportation system, maritime traffic pattern, AIS, trajectory data mining, trajectory clustering

## Abstract

Maritime traffic pattern recognition plays a major role in intelligent transportation services, ship monitoring, route planning, and other fields. Facilitated by the establishment of terrestrial networks and satellite constellations of the automatic identification system (AIS), large quantities of spatial and temporal information make ships’ paths trackable and are useful in maritime traffic pattern research. The maritime traffic pattern may vary with changes in the traffic environment, so the recognition method of the maritime traffic pattern should be adaptable to changes in the traffic environment. To achieve this goal, a dynamic maritime traffic pattern recognition method is presented using AIS data, which are cleaned, compressed, partitioned, and clustered online. Old patterns are removed as expired trajectories are deleted, and new patterns are created as new trajectories are added. This method is suitable for processing massive stream data. Experiments show that when the marine traffic route changes due to the navigation environment, the maritime traffic pattern adjusts automatically.

## 1. Introduction

Maritime traffic pattern recognition is used in obtaining a ship’s behavior pattern, such as route, mooring, and berthing area. A ship’s behavior is affected by many factors. Obtaining accurate results using only surveys and data collection is difficult. With the development of communication, computer, and network technology, a ship’s trajectory can be easily obtained from marine sensors. These sensors work all day, producing considerable heterogeneous multisource data with considerable spatial and temporal characteristics. The gathering and extraction of expected or unexpected valuable information from large-scale dynamic, vague, complex, abstract, or even confused data to judge and predict the behavior has become a research focus.

**Figure 1:**
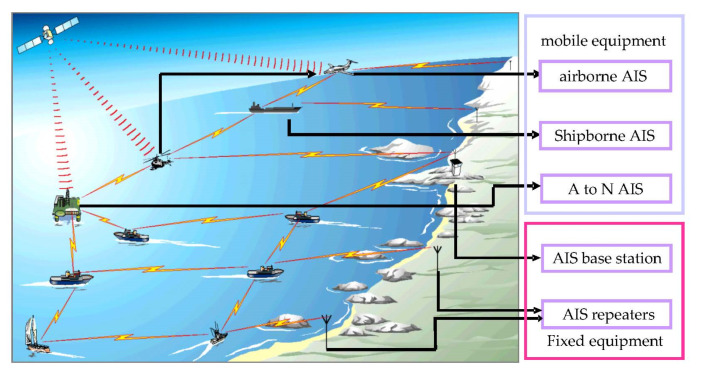
AIS communication link.

An automatic identification system (AIS) is an automatic tracking system used on ships and vessel traffic services (VTS) to identify and locate vessels. Vessels’ information can be exchanged among other nearby ships, AIS base stations, and satellites (Figure 1). According to the International Telecommunication Union specifications, AIS data include ship-related static and dynamic information. The static information, which is manually entered and automatically broadcast every six minutes or upon request, mainly includes the identification, ship name, type, call sign, and dimensions, among others. The dynamic information, which is from the Global Navigation Satellite System, and is broadcast at a time interval from two seconds to six minutes, depending on the vessel’s motion, includes the ship’s position (longitude and latitude), timestamp, course over ground, speed over ground, and true heading, among others. Ships fitted with AIS transceivers can be tracked by AIS base stations located along the coast lines or, when out of range of terrestrial networks, through a growing number of satellites fitted with special AIS receivers. According to the mandatory requirements of the Convention on the Safety of Life at Sea in 2002, all ships of 300 gross tonnage and upward engaged on international voyages, cargo ships of 500 gross tonnage and upward not engaged on international voyages, as well as passenger ships, irrespective of size, should be fitted with an AIS. With the widespread use of AIS base stations and satellites, a ship’s dynamic information can be easily obtained, facilitating marine scientific research and engineering applications.

In marine scientific research using AIS data, the following three aspects are focused on: marine risk analysis, anomaly detection, and maritime traffic pattern recognition. In marine risk analysis, Mazaheri, et al. [1] used statistical dependency to analyze the relationship between the characteristics of marine traffic (density and distribution) and the frequency of grounding accidents. Fixed and irregular grids were used to analyze marine traffic situations, aid the understanding of ship navigation patterns, and give the necessary input to assist in marine risk prediction [2,3,4,5]. Shu, et al. [6] used statistical methods to analyze the relationship between external factors (wind and visibility) and vessel behavior.

With artificial intelligence and big data development, marine anomaly detection, one of the main tasks for Marine Situational Awareness (MSA), is a recent research focus [7]. Vries and Someren [8] used DTW as the distance measure between trajectories. Kernel k-means, SVM, and one class SVM are used in clustering, classification, and abnormal behavior identification, respectively. Pallotta, et al. [9] used a density-based algorithm to cluster ship routes. KDE and Bayes rule were used to estimate the density function of clusters and identify abnormal behavior, respectively. Ristic, et al. [10] used adaptive KDE to cluster ship routes and proposed a probability method to detect anomalous behavior. Laxhammar [11] used the Gaussian Mixture Model (GMM) as a cluster model, and a greedy version of the Expectation-Maximization was used as the training algorithm. Laxhammar, et al. [12] compared the performances of the GMM and KDE in ship anomaly detection. Mascaro, et al. [13] used a Bayesian network to identify a ship’s anomalous behavior. In addition, ship trajectory clustering plays an important role in shipping network extraction [14], maritime traffic knowledge discovery [15], vessel trajectory prediction [16], ship path planning [17] and so on.

Maritime traffic patterns must be recognized to analyze marine traffic risk and identify vessel anomalous behavior. As clustering is an unsupervised approach that does not require prior knowledge, it is often used in the study of mobile pattern analysis [18]. The density-based method can group clusters in any shape, so many researchers favor it. According to the type of input sample, the method can be divided into clustering using points and clustering using trajectories [19]. The attributes of points are used for clustering, such as the location, course or speed. Lee, et al. [20] used time and date, position, SOG, and COG details as variables, and DBSCAN was used to identify the stationary points and turning points. Zhang, et al. [21] used the ship position, direction in and direction out as sample attributes, and DBSCAN was used to generate turning nodes. In addition, methods based on statistics [22], HDBSCAN [23] and OPTICS [24] have been proposed. Clustering using points conforms to the distance measure requirements of the basic theory of the clustering algorithm, and can be easily used in existing clustering algorithms. However, this method is easily influenced by local attribute changes; for example, collision avoidance behavior of ships may lead to a different result. So, clustering using trajectories was proposed. Trajectory similarity measure is used to compute the distance between trajectories for clustering. Trajectory similarity measure design is a special problem for this method. Dynamic time warping [25], Edit Distances [8], Hausdorff distance [26], Fréchet distance [27] and longest common sub-sequence (LCSS) [28] have been used as the trajectory similarity measure. Sheng and Yin [29] proposed a structural similarity measure considering speed, course, and position, which was used as the distance measure of DBSCAN to cluster, and inferred the optimal route using the Ant Colony Algorithm.

In the study of a ship’s behavior, maritime traffic pattern recognition is a fundamental problem that needs to be solved. The above studies have proposed their own solutions, but they did not explicitly consider the impact of environmental change on the existing patterns or prove that the patterns were still feasible after environmental changes, such as sea building, shipwreck, and waterway construction, among others. If a pattern fails to adjust to environmental changes, it will lose its practical application and cannot achieve fully automatic MSA. Consequently, a maritime traffic pattern dynamic recognition method is proposed. The method preprocesses the trajectory online, including trajectory cleaning, simplification, and partition. An improved LCSS distance is invoked as the similarity measure of the incremental DBSCAN to recognize the maritime traffic pattern.

The remainder of this paper is organized as follows: Section 2 introduces the method, Section 3 the effectiveness of the method, Section 4 discuss the results. The study’s conclusions are outlined in Section 5.

## 2. Methods

The program of Dynamic Maritime Traffic Pattern Recognition includes Trajectory Preprocessing and Trajectory Clustering. Trajectory Cleaning, Compression and Partition belong to Trajectory Preprocessing. All methods must be performed in an online mode.

AIS data need to be preprocessed before clustering. Preprocessed tasks include trajectory cleaning, simplification, and partition. First, some problems, such as wrong setting of the AIS transmitter, equipment failure, or weak information lead to a chaotic trajectory, position error, and signal interruption. If the trajectory is not cleaned, the experimental results would be severely affected. Second, because the AIS data size is large and contains many redundant points, if redundant data are not deleted, the system bears expensive data storage costs and time-consuming calculations. One of the best solutions is compressing AIS data using the trajectory simplification algorithm. Last, a ship may anchor, berth, enter, or leave the monitoring area according to the navigation plan. If the whole trajectory is used to cluster, many conventional local patterns would not be recognized. Particularly as some ships may sail in the harbor for a long time, in which case the AIS data would be received continuously, leading to memory overflow and clustering inability. Thus, a trajectory needs to be partitioned into several meaningful sub-trajectories for clustering.

The aim of trajectory clustering is to identify the common traffic pattern in a dynamic environment. The new trajectories can preserve or generate the existing traffic patterns and the old patterns can be deleted due to environmental changes. Therefore, the trajectory clustering algorithm should be able to quickly merge the new trajectories into existing traffic patterns or create new ones. Meanwhile, to improve the cluster speed and adapt to environmental changes, expired trajectories should be deleted and the traffic patterns adjusted accordingly. To achieve this goal, an effective incremental cluster algorithm is needed to perform the ship trajectory cluster, and the algorithm must include the functions of trajectory addition and deletion.

### 2.1. Trajectory Preprocess

#### 2.1.1. Trajectory Preprocessing Structure

**Figure 2:**
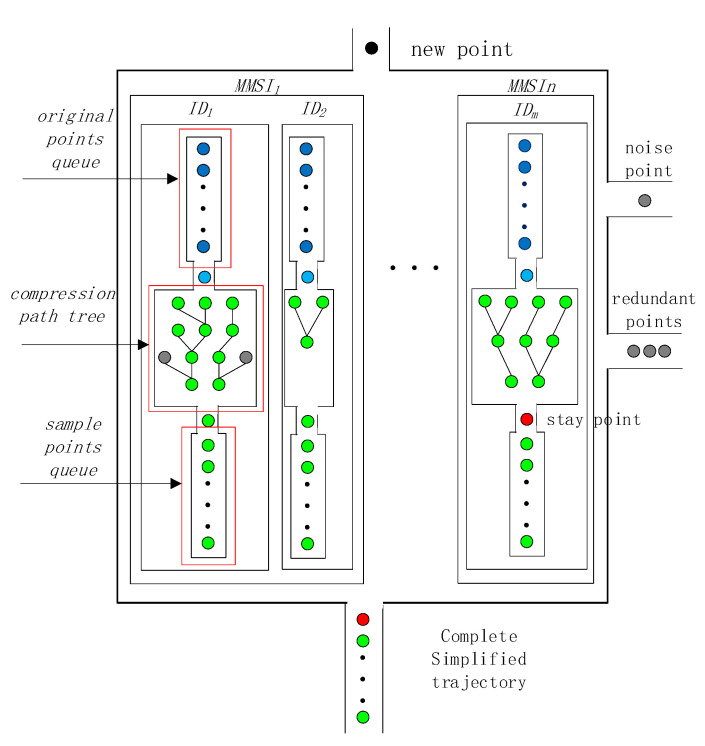
Trajectory preprocessing structure. MMSI is used as the unique identification code to exchange data. ID is used to identify a single trajectory. The original points queue is used to store the trajectory points, noise points are removed at this stage. The compression path tree is used to output the simplified trajectory, redundant points are removed at this stage. The sample points queue is used to store the simplified trajectory, when a stay point is detected, a complete simplified trajectory is output for clustering. The black circle represents a new received trajectory point. The dark blue circles represent the points ready to be cleaned. The green circles represent the sample points of the simplified trajectories. The gray circles represent noise points or redundant points. The red circles represent the stay points.

In order to implement the online preprocessed work, we designed a ship trajectory preprocessing structure (Figure 2). The structure contains three parts: the original points queue, the compression path tree, and the sample points queue. The original points queue is responsible for storing uncompressed points and cleaning the trajectory. The compressed path tree describes the shortest path through which each point can reach the first point, and it can be constructed using the trajectory simplification algorithm. The sample points queue stores sample points used to form simplified trajectories [30]. When a complete simplified trajectory is formed, it is output to the cluster algorithm. The details of these three parts are given in Section 2.1.2, Section 2.1.3 and Section 2.1.4.

#### 2.1.2. Trajectory Cleaning

An AIS transmitter uses a marine mobile service identification (MMSI) number as the unique identification code to exchange data. Each ship should have only one unique MMSI number. However, because the MMSI number is set manually, different AIS transmitters may have the same MMSI number. If directly linked, the points with the same MMSIs would cause trajectory confusion. In addition, due to equipment failure, a ship’s position might experience error, which would make the trajectory rough. Therefore, trajectory cleaning mainly solves two problems: MMSI sharing and position error.

1. MMSI sharing

In order to address this problem, a hash table stores the trajectory data set with the same MMSI. The hash table key is MMSI, and the value is a trajectory data set. A trajectory data set contains one or more trajectories. Each trajectory is constructed using several continuous points, stored in an original points queue, and set with a unique ID number. When a new point is received, the queue with the same MMSI and the distance and time span between the last point in the queue and the new point less than a certain threshold should be identified. When identified, the new point is inserted into the end of the first found queue. Otherwise, a new queue should be created. According to Recommendation ITU-R M.1371, the update interval of dynamic information is related to a ship’s dynamic conditions, with a nominal reporting interval from 2 s to 3 min, and the corresponding ship’s speed is from 2 knots to 23 knots. Considering possible signal loss, the signal could be lost three times. A ship with a speed of 3 knots could travel the farthest distance after losing the signal three times, with the corresponding update interval and maximum distance of 10 min and 0.5 nm, respectively.

2. Position error

**Figure 3:**
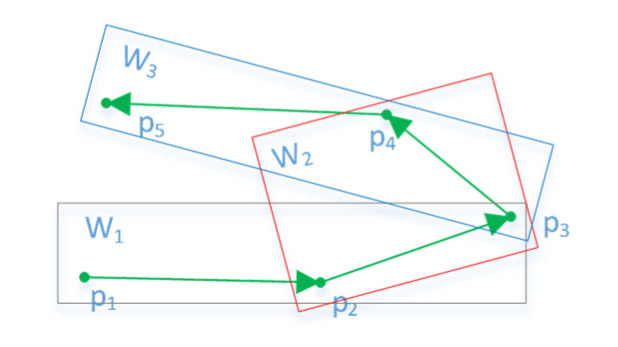
Multiple moving interpolation smoothing. A trajectory is composed by fine line segments, the green arrows represent their direction, five green dots are the sample points of the trajectory. Black, red and blue boxes are three sliding windows, each window contains three points. Three sliding windows are used to determine whether *p*_3_ is a noise point.

The AIS position is never accurate, due to GPS noise and other factors. If the noise is not filtered, the experimental results would be affected. Due to the large inertia of the ship, the ship’s trajectory should be a continuously changing process, and the abnormal jump point could be considered a noise point. Based on this idea, we designed a multiple moving interpolation smoothing method to delete abnormal points. There are five points (*p*_1_–*p*_5_) and three moving windows (*W*_1_, *W*_2_, and *W*_3_) (Figure 3). Each window contains three points. In each window, the position of the first and third points, and the time of the three points, are used to interpolate the position of the intermediate point. If the difference between the interpolations of a window and the received values is greater than the preset threshold value, one is added to the noise count of *p*_3_. When the noise counts of *p*_3_ are equal to three, it can be deleted as noise. The first two points and the last three points of the entire trajectory are deleted if they increase the noise count in the calculation; otherwise, they are retained. The use of multiple interpolation, rather than one interpolation, is to prevent normal points from being deleted as noise when encountering points with large changes in course or speed, such as *p*_3_. In this paper, the position threshold was set to 50 m.

#### 2.1.3. Trajectory Simplification

Simplification algorithms can be classified as either offline or online algorithms, based on whether they require the availability of the full data series [31]. An offline algorithm reduces the size of the trajectory after the trajectory has been fully generated. The DP [32] algorithm, as a typical offline algorithm, is often used to compress AIS data [33,34,35,36]. The online algorithm compresses a trajectory instantly, as an object travels. Typical online algorithms include [37,38,39,40,41,42]. Among them, the DOTS algorithm [42] uses Synchronized Euclidean Distance (SED) as a distance error and incremental directed acyclic graph (DAG) to realize online trajectory compression. SED considers the time characteristics of the original trajectory. DAG can generate a top-down compressed path tree. In the *V*_*k*+1_ layer construction of the tree, the new point chooses the point in the *V*_*k*_ layer whose Local Integral Square Synchronized Euclidean Distance is less than the threshold as its parent node. If more than one point satisfies this condition, the minimum Integral Square Synchronized Euclidean Distance should be chosen as the parent. If only one point in the top layer is attached to the bottom layer, the point is output to the simplified trajectory as a sample point. The DOTS algorithm can get the shortest compression path with minimum synchronize position error online and is used to simplify the AIS trajectory in this paper.

#### 2.1.4. Trajectory Partition

**Figure 4:**
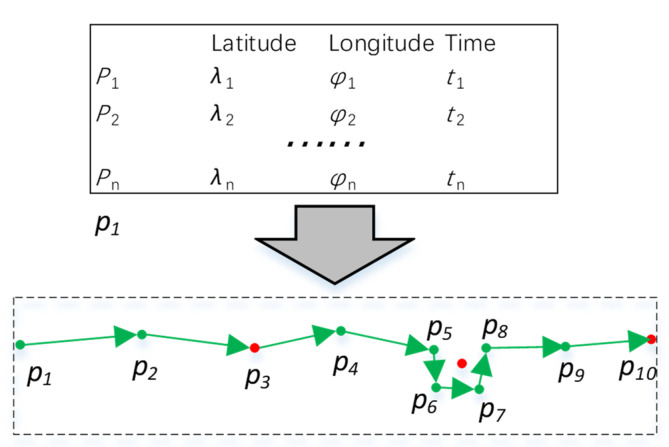
GPS records and stay points. A trajectory is composed by some line segments, green arrows represent their direction, the green dots are the sample points of the trajectory. The red dots represent the identified stay points.

A weak signal or closed AIS transmitter leads to trajectory interruption. Additionally, during a voyage, a ship may anchor, berth, and use different channels to enter and leave a port. In order to classify a ship’s behavior in different periods, trajectories need to be partitioned. In this paper, stay points were used to partition trajectories. Stay point stands for a geographic region where a ship stayed for a while. There are two categories of stay points (Figure 4). In one situation, like *p*_3_ and *p*_10_, a ship remains stationary for a period exceeding a threshold. In most cases, this happens when the ship closes the AIS transmitter, experiences a weak signal, or goes out of the study area. In the other situation, like *p*_5_–*p*_8_, a ship wanders around within a certain spatial region for a period. At this moment, several points are involved in the spatial region. Consequently, the mean coordinates of these points are used instead of the stay point. In most cases, this situation occurs when the ship is berthed or anchored.

In the stay point detection algorithm, two thresholds, distance and time span thresholds, need to be set. If a ship stops, the speed is approximately 0 knots, and the signal update interval is longer. Considering that the signal may be lost and the ship moves under the influence of wind and current, if the trajectory of a ship is not updated for more than 30 min, a stay point is obtained. In addition, if some points of a ship appear within 50 m for more than 30 min, these points are set as an alternative stop point set, and if the nearest distance and time between two alternative stop point sets are within 50 m and 30 min, they are merged into an alternative stop point set. The average position of an alternative stop point set is considered to be a stay point. When a stay point is detected, previous points can form a trajectory, which is then sent to the clustering algorithm.

### 2.2. Trajectory Clustering

#### 2.2.1. Similarity Measure

The determination of the similarity measure is key for the trajectory clustering effect. Two similar trajectories can be understood as the vertical distance from all points on one trajectory to the other trajectory satisfying a certain threshold, and the total length of the two trajectories is approximately equal. Armed with this idea, an improved LCSS was used for the trajectory similarity measure. The LCSS [43] distance was used to obtain the longest common sub-sequence existing in two trajectory sequences. For two point-based trajectories represented by $A=\left\{a_{1},\ldots ,a_{n}\right\}$ and $B=\left\{b_{1},\ldots ,b_{n}\right\}$, their LCSS distance was calculated as follows:

(1) $$L\left(A,B\right)=\{\begin{array}{c} 0,\;\;\;\;\;\;\;\;\;\;\;\;\;\;n=m=0\\ 1+L\left(Head\left(A\right),Head\left(B\right)\right),\;\;\;dist\left(a_{i},b_{j}\right)\leq \varepsilon \\ max\left(L\left(Head\left(A\right),B\right),L\left(A,Head\left(B\right)\right)\right),\;others \end{array}$$

where *L(A*,*B)* is the longest common sub-sequence of two trajectory segments *A* and *B*, the lengths of which are *n* and *m*, respectively. Head(A) and Head(B) represent the trajectory segments of *A* and *B* after removing their first track points. The value $dist\left(a_{i},b_{j}\right)$ represents the geographical distance between track points $a_{i}$ and $b_{j}$, *ε* is the distance threshold. When the distance difference between two trajectories *A* and *B* is less than *ε*, the pair of trajectory points are considered similar, and the value of *L* is increased by 1. If the number of points of trajectory *A* and *B* are both 0, then L(A,B) is 0. When the number of trajectory points is not 0, the maximum common sub-sequence can be obtained using Formula (1) recursively. The value *ε* determines the distance range of two trajectories to form a cluster, and its value should not be too large or too small. A large value merges different classes into one class, and a value that is too small splits the trajectories into too many fragmentary classes. Here, the value of *ε* according to the ship’s traffic flow width was determined using the traffic flow chart. The traffic flow width was measured on a monthly traffic flow chart between 1 and 3 nm. To improve the clustering resolution, *ε* was set to 1 nm.

**Figure 5:**
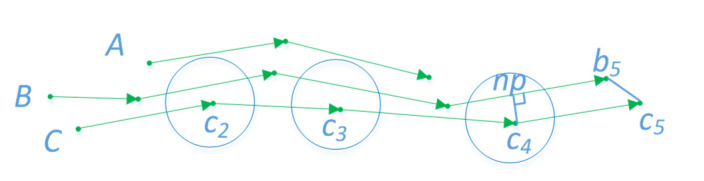
*A*, *B* and *C* are three trajectories, they are composed by several line segments and sample points. The green arrows represent the direction of the line segments, the green dots represent the sample points. Under the LCSS measure, *A* and *B* are similar, while *B* and *C* are dissimilar.

*L* can attain the maximum number of points between two trajectories satisfying *ε*, but the direct use of Formula (1) as the trajectory similarity measure leads to two problems (Figure 5). In the first situation, for different reasons, AIS generates a lot of debris trajectories, with differing total lengths of each trajectory. Like *A* and *B*, they are just similar in the middle part. If the short trajectory is similar to many long trajectories, it makes many dissimilar long trajectories gather into a cluster. In another situation, such as *B* and *C*, $C=\left\{c_{1},\ldots ,c_{n}\right\}$. Because the trajectories used to cluster are simplified, the distance between two continuous sample points is greater than the original. Intuitively, the two trajectories are similar, but points on one trajectory cannot find points nearby on another trajectory. Consequently, the two trajectories cannot be clustered together. To solve these two problems, the following improved LCSS equation was given:

(2) $$L^{*}\left(A,B\right)=\{\begin{array}{c} 0,\;n=m=0\;or\;\left|len\left(A\right)-len\left(B\right)\right|<\eta max\left(len\left(A\right),len\left(B\right)\right)\\ 1+L\left(Head\left(A\right),Head\left(B\right)\right),\;\;dist^{*}\left(a_{i},b_{j}\right)\leq \varepsilon \\ max\left(L\left(Head\left(A\right),B\right),LCSS\left(A,Head\left(B\right)\right)\right),\;others \end{array}$$

where *len*(*A*) and *len*(*B*) represent the total distance of *A* and *B*, respectively. The value *η* is a coefficient between [0, 1], meaning that two similar trajectories should have an approximately equal total length. Depending on experience, *η* was set to 0.2; that is, the total length difference between the two trajectories is no more than 20%. This condition can resolve the first problem. In the distance calculation, $dist^{*}\left(a_{i},b_{j}\right)$ represents the distance between the sample point and the nearest point of another trajectory. The nearest point can be the foot of the perpendicular or the endpoint on the line. For example, in Figure 5, *np* and *b_5_* are the nearest points of *c*_4_ and *c*_5_ on line *B*, respectively. This condition can resolve the second problem.

The value of *L* is unbounded and depends on the length of the compared sequences. To normalize it and support sequences of variable length, [43] proposed another distance measure based on LCSS. It can be changed to the following equation:

(3) $$D^{*}\left(A,B\right)=1-\frac{L^{*}\left(A,B\right)}{min\left(n,m\right)}$$

where min(n,m) represents the minimum value of n and m. This formula converts the *L* between trajectories to the distance between [0, 1].

#### 2.2.2. Incremental Clustering

**Figure 6:**
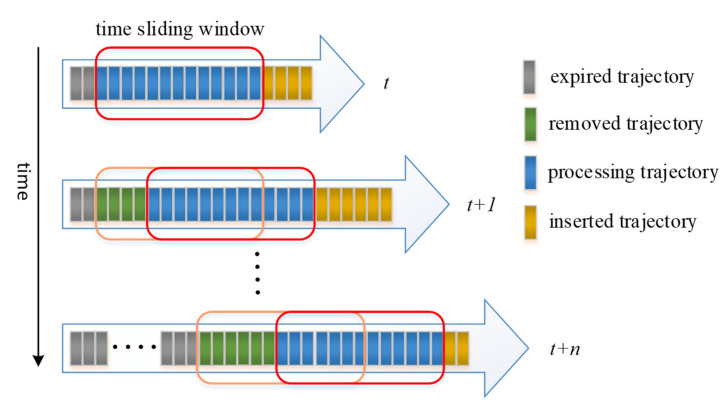
Continuous trajectories stream using sliding window model. Each cell represents a trajectory. The gray cells are the expired trajectories that have been removed from the cluster. The green cells are the removed trajectories that will be removed from the current cluster. The blue cells are the processing trajectories that have been absorbed to the current cluster. The yellow cell are the inserted trajectories that will be absorbed to the current cluster. The width of the sliding window represents the length of time. Trajectories within the sliding window are the dataset to generate the current cluster.

In this paper, the incremental DBSCAN [44] algorithm was used for trajectory clusters. The incremental DBSCAN is an efficient algorithm based on DBSCAN for mining data with density-based connectively [45]. The key idea of DBSCAN is that for each object of a cluster, if the neighborhood of a given radius (Eps) contains at least a minimum number of objects (MinPts), a density region is formed. These density regions can be connected by density-reachable objects. Owing to the density-based nature of DBSCAN, the insertion or deletion of an object affects the current clustering only in the neighborhood of this object. Thus, in an insert-or-delete operation, the incremental DBSCAN algorithm can get seed objects of affected objects, that is, objects that may potentially change cluster membership after the update. There are four possible cases when inserting a new object *p*: *p* is noise, a new cluster is created, *p* and some possible noise objects are absorbed into the cluster, or several clusters and the object *p* are merged into one cluster. There are three possible cases when deleting an old object *p*: *p* is deleted directly, some objects may become noise, or the deletion of *p* may cause a potential split. The incremental DBSCAN algorithm can dynamically maintain the clustering state and yields the same result as the DBSCAN. To distinguish the newly generated trajectory from the expired trajectory, a fixed time window width (TWW) was set to slide along the time axis. A small square represents a trajectory (Figure 6). On the whole-time axis, the trajectory is divided into the expired trajectory, the removal trajectory, the processing trajectory, and the inserted trajectory, according to the position of the time window. The expired trajectory represents data that has been removed from the clustering algorithm. The removal trajectory represents data that has expired but has not yet been removed. The processing trajectory represents data that has been clustered. The inserted data represent data that have not yet been clustered.

#### 2.2.3. Parameter Determination

In the incremental DBSCAN algorithm, there are three important parameters that need to be set: Eps, MinPts, and TWW. Eps is the distance threshold of similar trajectories, MinPts is the minimum number of trajectories to form clusters, and TWW is the time window width used to maintain the cluster state. The setting method of the three parameters is outlined below.

1. Setting of Eps

The trajectory distance can be obtained by Equation (3). When $D^{*}\left(A,B\right)<$Eps, the two trajectories are similar. Due to the large volume trajectory data and usual navigation on customary routes, a small Eps setting does not affect the formation of trajectory patterns. On the contrary, if the Eps is set too large, some trajectories that do not belong to one cluster will be merged into one. Eps was set to 0.1 in this paper; that is, 90% of the short trajectory points could find the nearest point on the long trajectory and they could group into a cluster. Through experiments, 0.1 was an appropriate setting value and could distinguish the trajectory pattern as far as possible and allowed for the existence of some noise points.

2. Setting of MinPts and TWW

There is interaction between MinPts and TWW. If MinPts is set too small, many debris trajectories are generated, and a stable maritime traffic pattern would not be formed. If MinPts is set too large, it increases the time for maritime traffic pattern formation. Therefore, the setting of MinPts should be the minimum TWW. The number of clusters could reach a relatively stable state. MinPts was determined by setting different MinPts and looking at the change in the number of clusters when TWW was infinite. Subsequently, the MinPts setting that made the number of clusters stable at the earliest time, and the length of time to reach this stable state, were selected as the setting of TWW.

## 3. Results

### 3.1. Experiments

**Figure 7:**
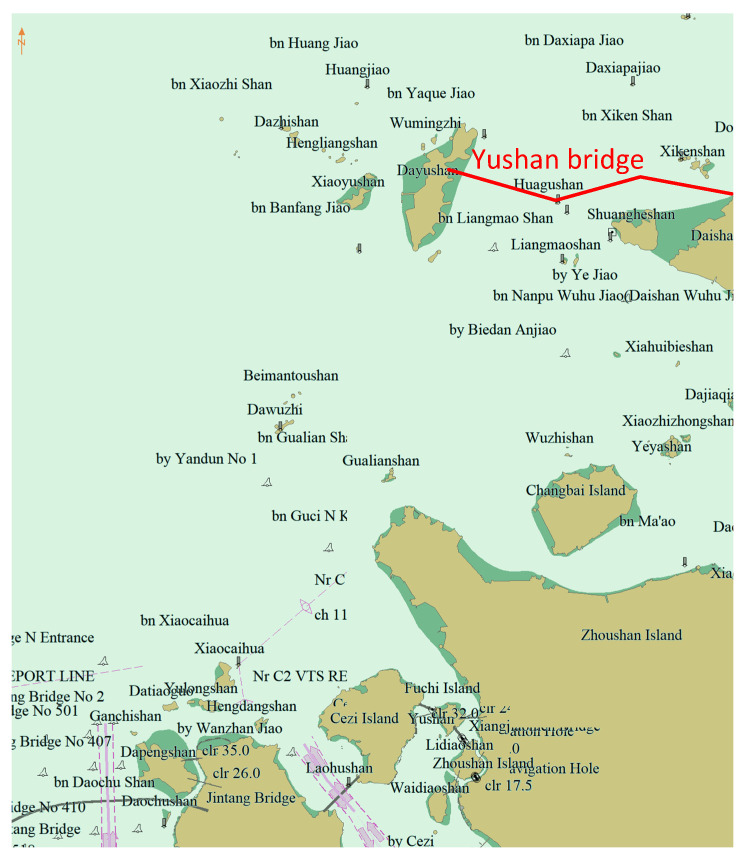
Experimental area and location of Yushan bridge. Yushan bridge is a newly constructed facility. During the construction of Yushan bridge, ships are banned from passing through the bridge.

To validate the efficiency and effectiveness of the proposed algorithm, AIS data from Zhoushan Port from June 2016 to December 2016 were used. The experimental range was between (30°01′ N, 121°43′ E) and (30°27′ N, 122°07′ E). The data were gathered through a network of AIS base stations set up by VTS along the Zhoushan coast of China. During this period, due to the construction of Yushan Bridge, from 20 September 2016 to 31 March 2019, ships were prohibited from passing through the bridge. This made the original traffic flow in the area disappear. The experimental area and the location of Yushan Bridge are given in Figure 7. Our experiment used the proposed algorithm for the cluster trajectory to see whether the cluster result could adjust traffic patterns with changes in the traffic environment.

### 3.2. Validation Results

**Figure 8:**
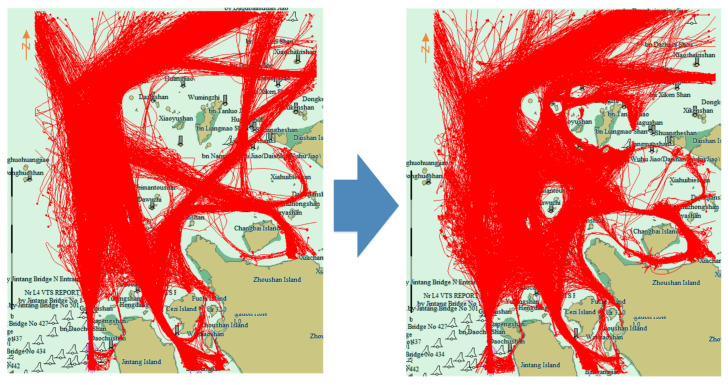
Traffic flow before and after prohibition of navigation. Red lines are the ship trajectories. Left picture is the traffic flow before the construction of Yushan bridge, right picture is the traffic flow after the construction of Yushan bridge. The traffic flow at Yushan Bridge was cut off after construction.

**Figure 9:**
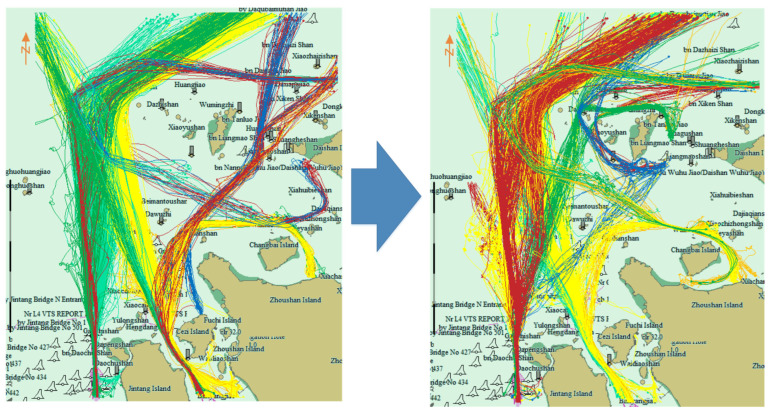
Clustering results before and after navigation prohibition. Different colors represent different clusters. After the construction of Yushan bridge, incremental clustering automatically makes the clusters across the bridge area disappear.

Figure 8 is the traffic flow chart of 40 days before and after 20 September 2016. On the left the first 40 days is illustrated, and on the right the last 40 days. It can be observed that the traffic flow passing through the bridge area disappeared after the prohibited time. Figure 9 shows the cluster results using the proposed algorithm before and after prohibition. Before the prohibition, there were 15 clusters, and after the prohibition, the number of clusters changed to 19.

**Figure 10:**
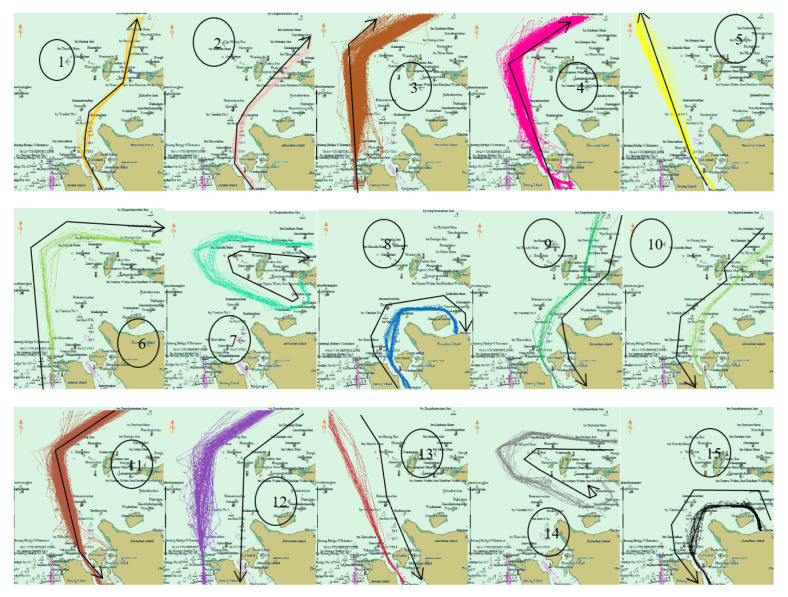
Cluster decomposition before navigation prohibition. There are fifteen clusters, black lines represent their shape and direction. Clusters 9 and 10 cross the bridge area.

**Figure 11:**
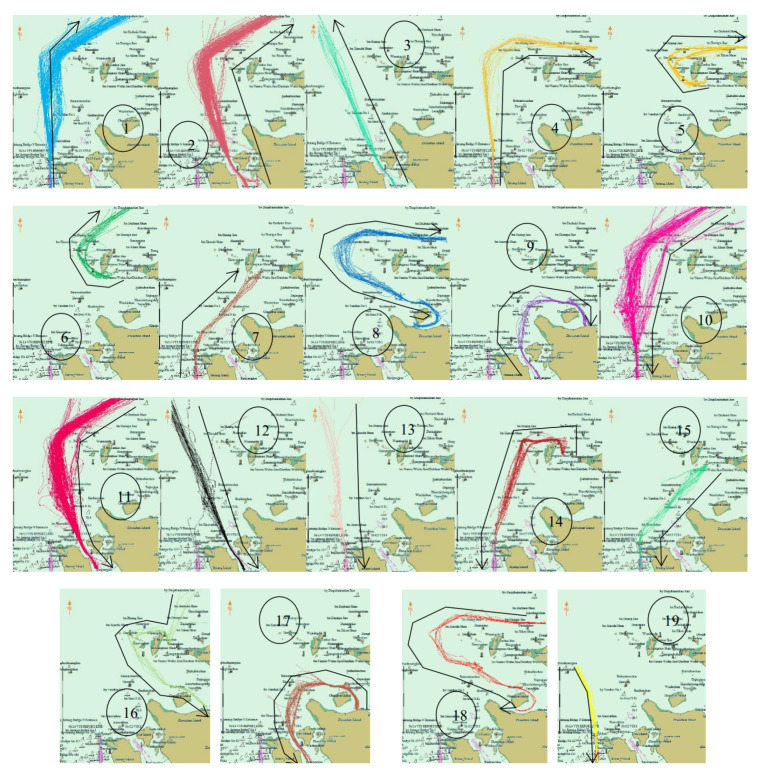
Cluster decomposition after navigation prohibition. There are nineteen clusters, the clusters crossed the Yushan bridge disappeared and some new clusters appeared.

Figure 10 displays the cluster decomposition before the prohibition. There were four clusters crossing the bridge. They were No. 1, 2, 9 and 10. However, after the prohibition, the traffic flow in the research area changed greatly. The original four clusters disappeared, and some new clusters appeared near the Yushan Bridge, such as No. 5–7 and 14–16 (Figure 11).

## 4. Discussion

### 4.1. Separation of Trajectories with the Same MMSI

**Figure 12:**
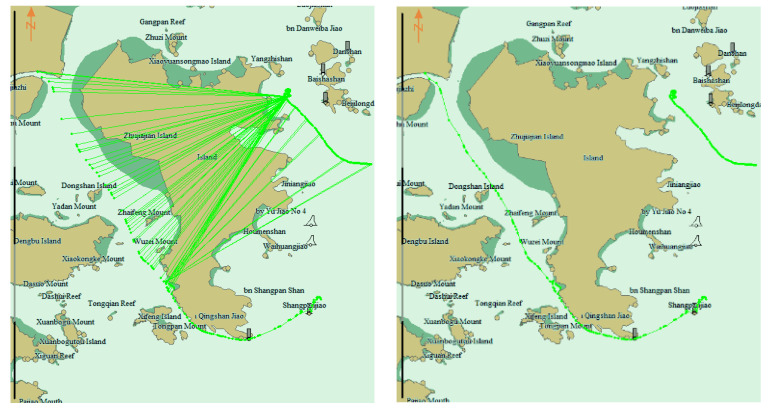
Clean result of two trajectories with the same MMSI. Left picture is the Uncleaned trajectories, the lines between the dots crossing land. Right picture is the cleaned trajectories, two trajectories are separated.

Figure 12 shows the clean result of two trajectories with the same MMSI. The picture to the left represents the original trajectory, with the points of the two trajectories alternately connected in chronological order. In the picture to the right, the two trajectories are separately displayed after processing.

### 4.2. Determination of MinPts in Clustering

**Figure 13:**
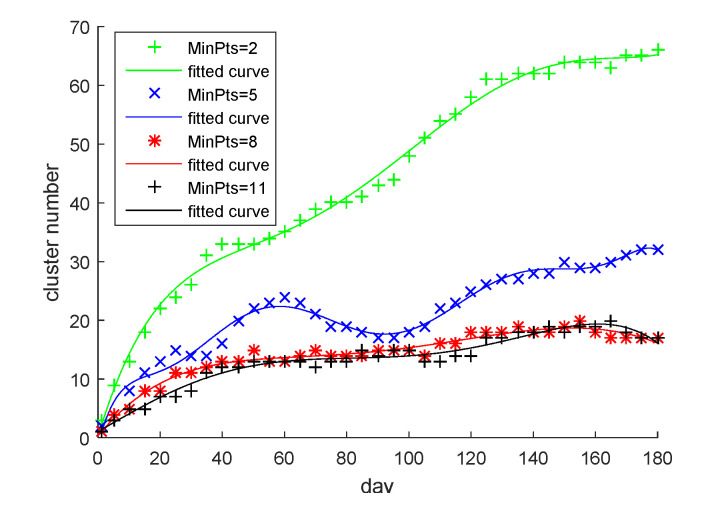
Dependence of the number of clusters with time for four MinPts settings. MinPts is the minimum number of trajectories to form clusters.

In this paper, we set four kinds of MinPts: 2, 5, 8, and 11. The total experiment time was 180 days. The number of clusters was recorded every five days. The curve fitted by the recording point is shown in Figure 13. When MinPts = 2, the number of clusters kept growing and did not reach a stable state. When MinPts = 5, the number of clusters fluctuated greatly, but began to show a stable trend. When MinPts = 8 and 11, the number of clusters could reach a stable state through a period of clustering, but the time for MinPts = 8 to reach a stable state, about 40 days, was shorter than that of MinPts = 11, which was about 60 days, after which the number of clusters was approximately the same. Therefore, MinPts = 8 and TWW = 40 days were the best values among the four sets. This setting not only ensured that the cluster number reached a relatively stable state after a period, but also minimized the time required to reach a stable state.

### 4.3. Comparison of Clustering Effect between Incremental DBSCAN and DBSCAN

In order to check the improvement of the incremental DBSCAN in the quality and speed of trajectory clustering, DBSCAN and incremental DBSCAN were used to cluster trajectories based on the same distance scale to see the cluster quality and speed.

**Figure 14:**
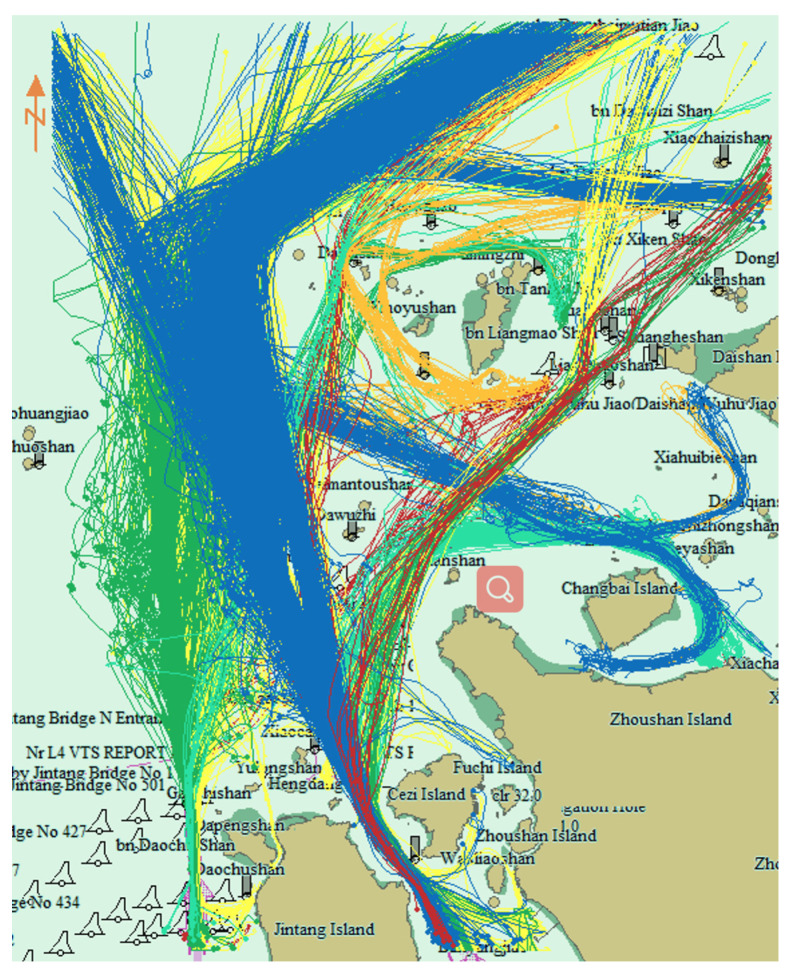
Cluster result using DBSCAN. After the construction of Yushan bridge, the clusters cross the Yushan bridge still exist, although no ships cross.

**Figure 15:**
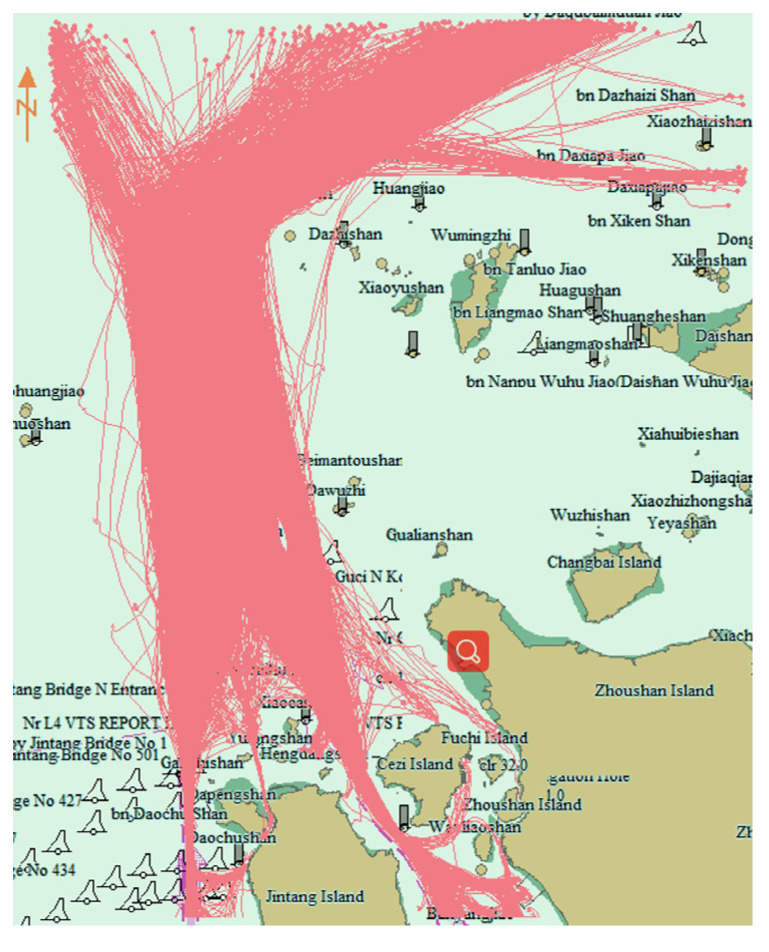
Independent trajectory merging result without deleting expired trajectories.

Regarding cluster quality, since DBSCAN did not delete the expired trajectories, clusters still existed after the prohibition of the channel of the Yushan Bridge (Figure 14). On the contrary, incremental DBSCAN could delete expired trajectories and clusters according to the TWW, so it would not keep the old traffic pattern all the time. Additionally, with the increase of noise trajectory, DBSCAN might merge irrelevant independent clusters. For example, the cluster in Figure 15 merged the cluster 3~6 (Figure 10) and the cluster 1–4 (Figure 11). It could be predicted that if the clustering time was long enough, more independent clusters would merge. Unlike DBSCAN, incremental DBSCAN deleted expired trajectories and only maintained the minimum number of trajectories reflecting current traffic patterns. It could not only adapt to the traffic pattern change caused by the traffic environment but could also reduce the impact of noise trajectory on the clustering result.

**Figure 16:**
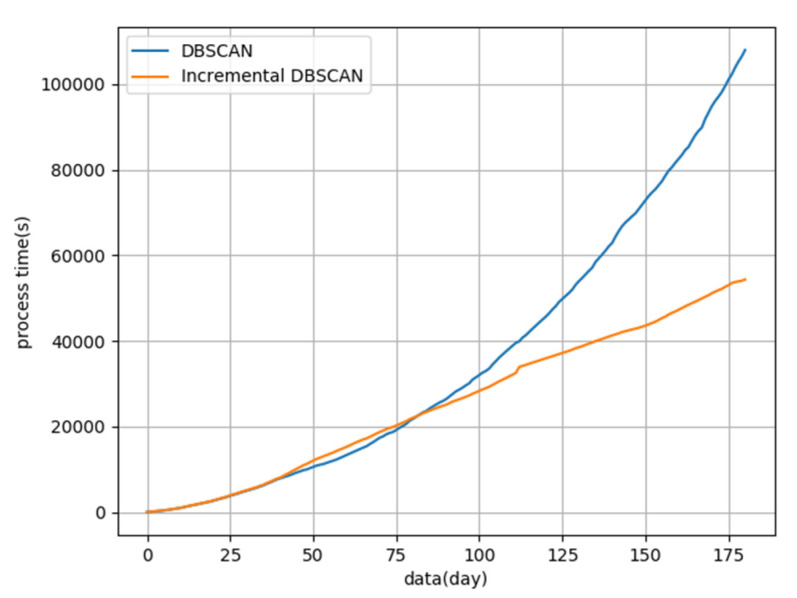
Comparison of clustering time between DBSCAN and incremental DBSCAN.

Regarding cluster speed, incremental DBSCAN was more efficient than DBSCAN with an increase in time. Figure 16 is a time-consuming comparison between DBSCAN and incremental DBSCAN. The first 40 days of clustering, the processing time of the two algorithms was almost equal. Between 40 and 80 days, the processing time of incremental DBSCAN increased slightly due to the addition of deletion programs. However, after 80 days, the processing time of DBSCAN was longer than that of incremental DBSCAN, and the gap between them widened. Overall, the processing time of the incremental DBSCAN increased almost linearly, while that of DBSCAN tended to follow an upward parabolic trend.

## 5. Conclusions

Maritime traffic pattern recognition is employed to obtain the conventional behavior of ships. The marine traffic environment is complex and changeable. The method of maritime traffic pattern recognition should be adaptable to changes in the traffic environment. Based on AIS data, a dynamic recognition method for maritime traffic patterns was proposed. This method is composed of two parts: trajectory online preprocessing and incremental clustering. The trajectory online preprocessing cleans, simplifies, and partitions the trajectory. Track cleaning can separate MMSI number shared trajectories and delete abnormal points. A DOTS algorithm is used to simplify the trajectory. The redundant points can be deleted on the premise of minimizing Synchronized Euclidean Distance error. The trajectory partition algorithm can divide a ship’s trajectory into several segments. The divided trajectory can reflect the local behavior characteristics of the ship. Regarding trajectory clustering, the improved LCSS is used as the trajectory similarity measure. The measure can solve the impact of sparse trajectory points and debris trajectory. Incremental DBSCAN is used to cluster trajectories. It can reduce the impact of noise trajectory on clustering results and improve the speed of large data trajectory clustering. To check the effectiveness of the proposed algorithm, the AIS data near Yushan, Zhoushan Port, China was used for the experiment. The experimental contents included the dynamic recognition of maritime traffic patterns and the comparison of pattern recognition quality and speed between DBSCAN and incremental DBSCAN. The experimental results showed that the proposed algorithm could meet the needs of maritime traffic pattern dynamic recognition with changes in the navigation environment, and clustering speed was more stable with the increase of data volume.

## Data Availability

Not applicable.
